# The challenge of evolving stable polyploidy: could an increase in “crossover interference distance” play a central role?

**DOI:** 10.1007/s00412-015-0571-4

**Published:** 2016-01-12

**Authors:** Kirsten Bomblies, Gareth Jones, Chris Franklin, Denise Zickler, Nancy Kleckner

**Affiliations:** Department of Cell and Developmental Biology, John Innes Centre, Norwich Research Park, Colney, Norwich, NR4 7UH UK; The Red House, St. David’s Street, Presteigne, Powys (Wales) LD8 2BP UK; School of Biosciences, University of Birmingham, Edgbaston, Birmingham, B15 2TT UK; Institut de Génétique et Microbiologie, I2BC, Université Paris-Sud, Orsay, France; Department of Molecular and Cellular Biology, Harvard University, Cambridge, MA USA

**Keywords:** Polyploidy, Meiosis, Crossover interference, Homologous chromosomes, Recombination, Chiasmata

## Abstract

Whole genome duplication is a prominent feature of many highly evolved organisms, especially plants. When duplications occur within species, they yield genomes comprising multiple identical or very similar copies of each chromosome (“autopolyploids”). Such genomes face special challenges during meiosis, the specialized cellular program that underlies gamete formation for sexual reproduction. Comparisons between newly formed (neo)-autotetraploids and fully evolved autotetraploids suggest that these challenges are solved by specific restrictions on the positions of crossover recombination events and, thus, the positions of chiasmata, which govern the segregation of homologs at the first meiotic division. We propose that a critical feature in the evolution of these more effective chiasma patterns is an increase in the effective distance of meiotic crossover interference, which plays a central role in crossover positioning. We discuss the findings in several organisms, including the recent identification of relevant genes in *Arabidopsis arenosa*, that support this hypothesis.

**Electronic supplementary material:**

The online version of this article (doi:10.1007/s00412-015-0571-4) contains supplementary material, which is available to authorized users.

## Introduction

Most eukaryotic organisms have diploid genomes. However, in some cases, a genome contains more than two homologous copies of each chromosome. This condition, known as “polyploidy,” occurs in many species, notably plants but also many animals (Astaurov 1969; Ramsey and Schemske 1998; Otto and Whitton 2000; Ramsey and Schemske 2002; Comai 2005; Soltis et al. 2007; Doyle et al. 2008; Grandont et al. 2013; Stenberg and Saura 2013; Ianzini et al. 2009). Polyploidy is a potent evolutionary force that is implicated in increases of genome complexity, adaptation, and speciation (Soltis et al. 2003; Rieseberg and Willis 2007; Fawcett and Van de Peer 2010; Arrigo and Barker 2012). However, when the polyploid condition first arises, it causes substantial problems for basic cellular processes, most notably for regular chromosome segregation during meiosis, the specialized cellular program that underlies gamete formation for sexual reproduction (Ramsey and Schemske 2002; Comai 2005; Stenberg and Saura 2013; below). Nonetheless, this problem can ultimately be resolved: stable, sexually reproducing polyploid lineages are found in nature and sometimes can be generated in experimental settings upon selection for fertility. How these states evolve remains largely mysterious, and investigation of this question promises intriguing insights into how fundamental processes can be evolutionarily modified without perturbing their essential functions.

Polyploids generally fall into two broad categories, albeit with a range of intermediate states (Ramsey and Schemske 1998; Otto and Whitton 2000; Bomblies and Madlung 2014). “Autopolyploids” arise by whole genome duplication within an individual or within a species, yielding genome complements in which all copies of a particular chromosome are closely homologous (although still carrying sequence differences present between homologs in the originating diploid). “Allopolyploids,” in contrast, arise by formation of hybrids with related yet distinct genomes, accompanied by whole genome duplication. The component genomes of allopolyploids thus comprise two or more sets of diploid genomes, with a closer homology between the two chromosomes of each set and a significantly less homology between chromosomes of different sets.

In diploid meiosis, homologs regularly segregate away from one another, to opposite poles, at the first division of meiosis (MI); sister chromatids then segregate at the second division of meiosis (MII). For full fertility in polyploid meiosis, the MI must still somehow result in a regular segregation of equal numbers of parental chromosomes to each pole, despite the fact that there are now multiple copies of each chromosome present. This challenge is particularly stark for autopolyploids, because the different versions of each chromosome are very similar or indistinguishable in DNA sequence and thus provide no special intrinsic cues to guide equi-partitioning during the segregation process. For allopolyploids, in contrast, the presence of homology can be (and is) used to solve this problem, as shown by the fact that more homologous chromosomes preferentially segregate from one another (e.g., Holm 1986; discussion in Zickler and Kleckner 1999; Bhullar et al. 2014; Martín et al. 2014). We focus here on the more dramatic case of autopolyploidy, which has been investigated extensively from various perspectives.

## Newly arising autotetraploids evolve to a stable fertile state via restrictions on the number and types of chiasma configurations

Newly emerged autopolyploid lines generated in the laboratory or arising in nature generally exhibit meiotic chromosome mis-segregation in meiosis I, resulting in the formation of aneuploid gametes and compromised fertility (Ramsey and Schemske 1998, 2002; Hilpert 1957; Santos et al. 2003; Comai 2005; Grandont et al. 2013; Sybenga 1975). However, naturally occurring polyploids can evolve solutions to overcome these problems.

### Diploid meiosis

To contextualize the challenges faced by polyploids in meiosis, we first discuss the normal process that occurs in diploids.

**Fig. 1:**
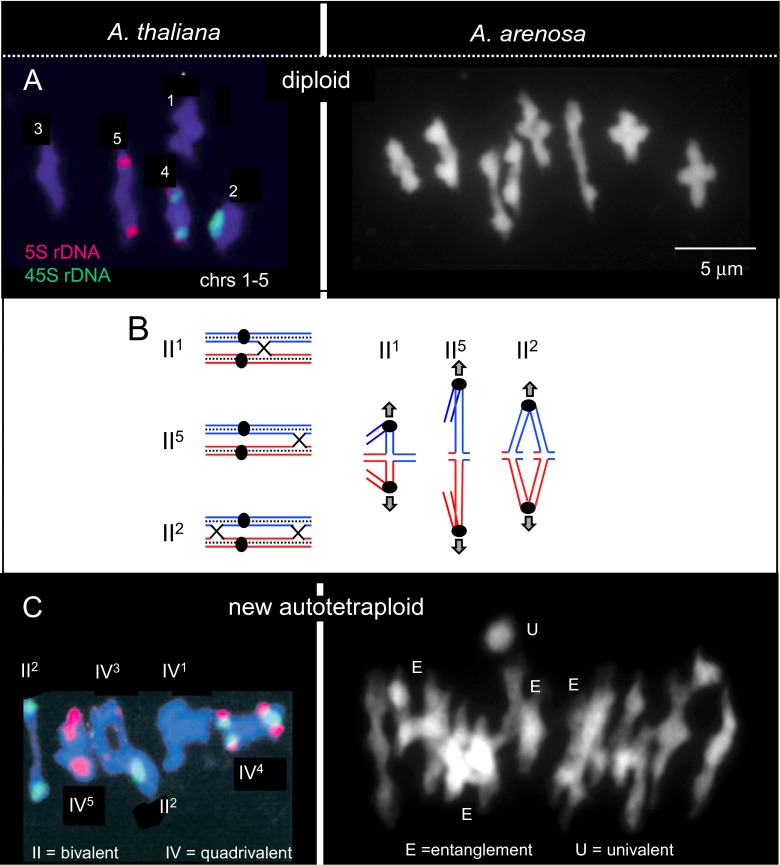
Metaphase I (MI) configurations in diploids and newly arisen autotetraploids of *Arabidopsis*. Chromosomes are being pulled towards opposite spindle poles (*above* and *below*, respectively) via attachments of microtubules to the respective centromere/kinetochore regions. *a* (*Left*) *Arabidopsis thaliana* diploid showing five bivalents (from López et al. 2012). The chromosome number of each bivalent is indicated. 45S and 5S rDNA loci are indicated. *a* (*Right*) *Arabidopsis arenosa* diploid showing eight bivalents (C.F. and C. Morgan, unpublished). *b* Cartoons showing chromosome associations that give rise to three of the metaphase I configurations seen in *a* (*left*). The *arrows* indicate the orientation of centromeres (*filled circles*) towards opposite spindle poles. *II* denotes the bivalent; *superscript* denotes the chromosome number. *c* (*Left*) An experimentally created autotetraploid of *A. thaliana* showing a mixture of bivalents (*II*) and quadrivalents (*IV*) (from Santos et al. 2003). *c* (*Right*) An experimentally created autotetraploid of *A. arenosa* showing some identifiable bivalents, many complex configurations in which multiple chromosomes are entangled (*E*) and one apparent univalent (*U*) (Chris Morgan and C. F., unpublished)

In a diploid cell at metaphase I, the centromeres of homologous chromosomes (homologs) are oriented towards opposite poles, in preparation for segregation during the ensuing anaphase (Fig. 1(a, b)). At this stage, homologs are connected to one another at specific positions, seen cytologically as one or more “chiasmata,” each of which results from the combined effects of a DNA crossover (CO) between homolog non-sister chromatids plus links between sister chromatid arms along their lengths (Fig. 1(a, b)).

These connections are essential for the regular segregation of homologs to opposite poles, analogous to the way in which a physical connection of sister chromatids ensures bipolar orientation and segregation during mitosis and metaphase/anaphase of the second meiotic division (Li and Nicklas 1997; Nicklas 1997). In all cases, connectedness is important because it allows centromere/kinetochore complexes to be placed under mechanical tension as the spindle is forming. This tension, in turn, has two effects which, together, ensure bipolar orientation of the segregating units (Li and Nicklas 1997; Nicklas 1997; Lampson and Cheeseman 2011). First, tension stabilizes microtubule/kinetochore attachments, thus locking each bivalent into the correct configuration. Second, tension signals to the spindle regulatory surveillance system (the “spindle assembly checkpoint”) that a bivalent is properly aligned and thus ready to be correctly pulled towards opposite poles during anaphase I. When all segregating pairs (e.g., meiotic homolog “bivalents”) are correctly oriented to opposite poles, cellular regulatory mechanisms trigger anaphase onset. In the absence of correct orientation, anaphase eventually proceeds but with a significant delay.

In accordance with these considerations, a first requirement for regular MI segregation is that each pair of homologs must (and does) acquire at least one CO (and thus chiasma). This feature is often called the “obligatory CO.” Many diploid species exhibit only one or two COs/chiasmata per bivalent (e.g., *Arabidopsis thaliana* and *Arabidopsis arenosa*; Fig. 1(a, b)); others exhibit larger numbers.

A second requirement for regular MI segregation is that each bivalent must be a separate physical unit that is free of entanglements with other bivalents. In fact, interlockings among unrelated chromosomes often arise during prophase as a consequence of the way in which homologs undergo recombination while becoming coaligned/paired and synapsed. However, interlocks are also concomitantly actively eliminated (Storlazzi et al. 2010). Thus, at MI, interlocked bivalents are rare or absent.

### Meiosis in neo-autotetraploids

A newly arisen autotetraploid line faces severe challenges during MI chromosome segregation. Since all four copies of each chromosome are essentially the same, COs can (and do) occur indiscriminately among these copies in all pairwise combinations. This situation compromises both of the features required for regular two-by-two segregation.

First, newly arising autotetraploids exhibit complex arrays of chiasma configurations (e.g., Fig. 1(c)). The four chromosome copies may happen to form two pairs of bivalents that will give regular two-by-two segregation. However, they can also form diverse types of multivalents (where more than two copies are connected).

**Fig. 2:**
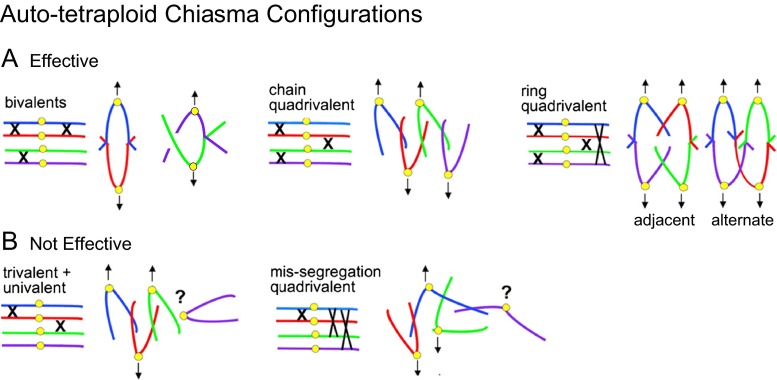
Chiasma configurations for an autotetraploid that are either effective for ensuring two-by-two segregation (**a**) or not (**b**). Effective segregation requires that each chromosome be linked to either one or two other chromosomes. Only three configurations satisfy this requirement. By contrast, if any chromosome (or more than one chromosome) is unlinked to a partner or is linked to all three other homologs, segregation will be aberrant, as illustrated for representative single chromosome cases

Many of the configurations that arise in neo-autotetraploids are incompatible with regular two-by-two segregation. Overall, the operative rule for effective two-by-two segregation is that every one of the four homologous chromosomes must be linked to at least one, but not more than two, partner chromosome(s) (Fig. 2). Sets of linkages that satisfy this rule comprise bivalents and either chain or ring quadrivalents (Fig. 2a). In all three cases, spindle tension is achieved by orientation of two centromere/kinetochore regions towards each pole as appropriate to two-by-two segregation. In contrast, any complement that includes a chromosome(s) unlinked to any partner will be ineffective (e.g., a univalent-plus-trivalent combination; Fig. 2b, left). Additionally, in any complement where one or more chromosomes are linked to each of the three other homologous chromosomes, one centromere will always be unable to come under tension and thus again will not reliably segregate to the appropriate pole (Fig. 2b, right).

Second, neo-autotetraploid chromosomes exhibit extensive numbers of chromosomal associations that appear to be comprised of both multivalents and entanglements (e.g., “E” in Fig. 1(c); see also Fig. 5 in Yant et al. 2013). These configurations are the consequence of the fact that all four homologous chromosomes often pair and synapse and form CO connections; moreover, if other homologs are trapped in those quadrivalents, they can remain entangled by their own CO connections up to metaphase I. In diploids, such entanglements (“interlocks”) are usually resolved before diplotene/metaphase I (von Wettstein et al. 1984). However, if they are not, the presence of entanglements is an obvious problem for MI segregation. In new autotetraploids, the presence and persistence of interlockings might primarily be responsible for the overall high mis-segregation frequencies as well for the occurrence of univalents. The resolution of interlocks requires, among other events, Mlh1-mediated release of trapped recombinational interactions (Storlazzi et al. 2010). Such a process would promote disassembly of CO-fated recombination complexes that are stalled at sites of interlocks. Given that CO complexes are relatively few to begin with, the result would be a tendency for some of the entrapped chromosomes to lose all COs, thus lacking even the single “obligatory” CO and appearing as univalent(s) at MI.

### Meiosis in evolved autotetraploids

Evolved autotetraploid species commonly show regular tetrasomic inheritance (Quiros 1982; Krebs and Hancock 1989; Wolf et al. 1989; Fjellstrom et al. 2001; Hollister et al. 2012): each locus segregates four alleles, with two of the four parental alleles randomly segregating into each gamete. Thus, evolution of an autotetraploid into a stable, sexually reproducing state has achieved regular two-by-two segregation without the emergence of specific genetically defined partner preferences. To do so, autotetraploid evolution includes changes in chiasma patterns.

First, there is a reduction in the total overall frequency of chiasmata. In general, well-evolved autotetraploids tend to have lower overall frequencies of COs than newly formed autotetraploids (e.g., Morrison and Rajhathy 1960a; Mulligan 1967; Reddi 1970; Yant et al. 2013; Wu et al. 2013). More specifically, stable autotetraploid lines that have evolved in nature have lower CO frequencies than the diploid lines from which they originated (Mulligan 1967; Yant et al. 2013). Furthermore, when an autotetraploid is created experimentally and then allowed to evolve under selective pressure for successful meiotic transmission, a decrease in multivalent frequency commonly occurs, associated with a reduction in the level of COs, which progressively decreases with the number of rounds of selection (e.g., Bremer and Bremer-Reinders 1954; Povilaitis and Boyes 1956; Hilpert 1957; Morrison and Rajhathy 1960a; Lavania et al. 1991; Santos et al. 2003). In a number of species, the frequency of chiasmata is reduced to one, thus nearly the minimum possible level. For example, autotetraploids *Lotus corniculatus*, *A. arenosa*, and *Physaria vitulifera* all average about 1.1 crossovers per bivalent (Mulligan 1967; Davies et al. 1990; Yant et al. 2013). Lower numbers of COs/chiasmata not only match the emergence of more restricted chiasma configurations (above) but also will tend to reduce the probability of persisting MI interlockings.

The only exception to this trend is that, in the few related grass species where autotetraploids exhibit mostly quadrivalents with terminal chiasmata (e.g., *Dactylis glomerata*; Table S1), the chiasma frequency is higher than that in the corresponding diploids or newly formed autotetraploids. Apparently, an evolved autotetraploid state with three or four chiasmata per quadrivalent emerged from a standard diploid condition with one or two chiasmata per bivalent.

**Fig. 3:**
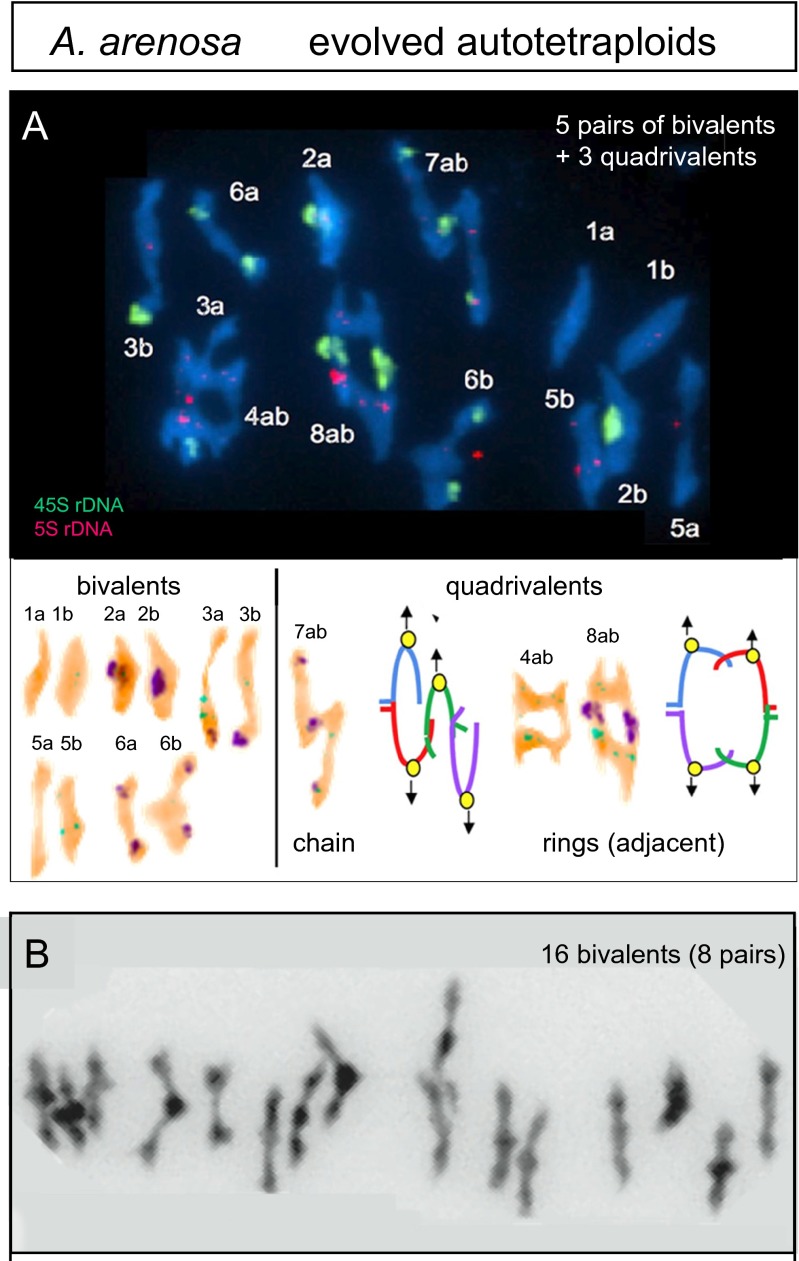
Two metaphase I complements for a fully evolved autotetraploid of *Arabidopsis arenosa* (C. F. and C. Morgan, unpublished). **a** A majority of bivalents plus a minority chain and ring quadrivalents (10 bivalents corresponding to 5 pairs plus 3 quadrivalents). Accompanying color-inverted images show chromosome constitution and multivalent configurations. **b** Full complement of 16 bivalents corresponding to 8 pairs

Second, MI chiasma configuration types become more restricted, being limited specifically to those that effectively promote regular two-by-two segregation. Thus, trivalent-plus-univalent combinations are rare in naturally evolved tetraploids and, in experimental evolution studies, occur with a decreasing frequency as a stable state is progressively achieved (Povilaitis and Boyes 1956; McCollum 1958; Hazarika and Rees 1967; Jones 1967; Santos et al. 2003). Furthermore, a survey of 20 naturally evolved autopolyploid species (Table S1) reveals 12 species that form exclusively or almost exclusively bivalents at MI, with each set of four chromosomes comprising a pair of bivalents; five other species show mostly bivalents, but also some quadrivalents (e.g., *A. arenosa*; Fig. 3a, b), and three related species show primarily or exclusively quadrivalents. The bivalent-plus-quadrivalent solution also emerges in the laboratory when a newly formed autopolyploid evolves into a more stable line by selection for fertility over a number of generations, with improvements sometimes seen after only a few generations of selection (Gilles and Randolph 1951; Bremer and Bremer-Reinders 1954; Hilpert 1957; Santos et al. 2003). Moreover, the quadrivalents seen in these evolved situations are either chains or rings (Fig. 3b), i.e., the two specific types that are effective for two-by-two segregation (Fig. 2a). In contrast, other possible quadrivalent configurations are rare, absent, and/or anti-correlated with regular segregation (e.g., McCollum 1958).

Ring quadrivalents can exhibit either of two MI segregation configurations according to whether “alternate” or “adjacent” centromeres are linked to the same pole (Figs. 2a and 3a). The former configuration is favored: as autotetraploidy evolves, the alternating ring configuration increases in abundance while the adjacent ring configuration decreases (McCollum 1958; Mosquin 1967). These preferences correspond to the dictate that spindle tension should be maximized: in rings of the favored alternate orientation, all four centromere/kinetochore complexes are under tension from both sides whereas, in the less-favored adjacent orientation, pairs of bi-oriented complexes are under tension from only one side.

Third, there is a tendency for modulation of chiasma position during autotetraploid evolution. There is no universal requirement for localization of chiasmata to particular positions. For example, in *A. arenosa*, the chiasmata in an evolved autotetraploid can occur at centromere-proximal, centromere-distal (“sub-terminal”), and interstitial positions as seen from MI configurations and confirmed by molecular analysis of CO-correlated Mlh1 foci along prophase chromosomes (Chris Morgan, C. F., and K. B., unpublished). On the other hand, the occurrence of chiasmata near chromosome ends (referred to as “terminal” localization) is positively correlated with regular quadrivalent segregation (Myers 1945; McCollum 1958; Hazarika and Rees 1967; Jones 1967), implying that such localization might be particularly helpful for creating the appropriate quadrivalent configurations. In another variation, some autotetraploid *Allium* species have centromere-proximal COs/chiasmata (Table S1).

Chiasmata are prominently terminal in autotetraploids of grasses and cereals (Hazarika and Rees 1967; McCollum 1958). However, the same tendency is also seen in the corresponding diploid lines. Perhaps earlier in their evolution, diploids became autotetraploids, which evolved terminal chiasmata, and then returned to the diploid state. Indeed, many apparently diploid genomes give evidence of prior polyploidization (e.g., Mitchell-Olds and Clauss 2002). More generally, terminal localization of chiasmata might facilitate ready interconversion between diploidy and autotetraploidy.

Fourth, evolved autotetraploids also lack the high levels of interlockings among unrelated chromosomes that characterize newly emerged lines, with resolution during pachytene as in the diploid case (compare Fig. 3a, b versus Fig. 1(c); Higgins et al. 2014a; Yant et al. 2013).

## Modulation of CO formation for autotetraploid evolution

What type of mechanism(s) might explain how newly formed autotetraploids evolve the specific chiasma configurations needed to support regular two-by-two MI segregation? Since CO positions are determined during prophase, evolutionary forces are presumably acting on events that occur during this period, long before chiasmata are actually required to mediate chromosome alignment and segregation.

### CO formation in diploid meiosis

Universally, meiosis involves the initiation of recombination via a large number of programmed double-strand breaks (DSBs) which interact primarily with homolog partners to give a large number of early recombinational interactions (Hunter 2006; Zickler and Kleckner 2015). A minority subset of these many interactions is then designated to eventually mature into COs (“CO designation”) with the remainder maturing to other fates. When a bivalent exhibits more than a single CO, those COs exhibit the classical feature of “crossover interference”: the presence of a CO at one position is accompanied by a reduced probability that another CO will occur nearby (Sturtevant 1915). The strength of this reduction decreases with increasing interposition distance. Importantly, DSB formation and all ensuing DNA events leading to CO formation occur in recombination complexes that are in direct physical and functional association with developing or developed axes (Kleckner 2006; Kleckner et al. 2011; Zickler and Kleckner 2015; Storlazzi et al. 2010). Correspondingly, it now appears that the “metric” for the interference effect is physical distance along the chromosome, e.g., along chromosome axes, with absolute distances ranging from 300 nm to many microns according to the organism, rather than either genomic distance (Mb) or genetic distance (cM) (discussion in Zhang et al. 2014b).

**Fig. 4:**
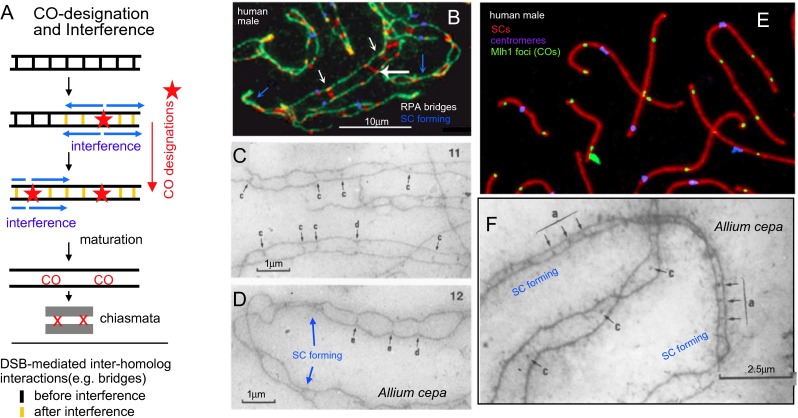
Prophase chromosomal events in diploid meiosis. **a** “Fill-in-the-holes” model for CO position selection (see also Wang et al. 2015). The array of early total DSB-mediated recombinational interactions (e.g., bridges; **b**–**d** (*below*)) is acted upon by a CO designation process. Each CO designation (*red star*) sets up an inhibitory zone of “CO interference” (*blue arrows*) via a signal that spreads outwards in both directions, dissipating with distance. This signal prevents bridge interactions in the affected region from undergoing CO designation (indicated by bridges changed to *yellow*). Subsequent CO designations occur in regions away from previously established interference zones, ultimately filling in the holes between previous CO sites. CO designation is very efficient, thus ensuring that all homolog pairs acquire at least one (first, obligatory) CO. **b**–**f** Homolog coalignment (“pairing”) is mediated by inter-axis bridges that comprise DSB-mediated recombinational interactions and followed by SC formation (“synapsis”). **b**, **e** Human male meiotic prophase chromosomes visualized by immunofluorescence illumination. **c**, **d**, **f** *Allium cepa* axes and associated “zygotene” recombination nodules (ZNs) or bridges (corresponding approximately to many/all DSB-mediated interactions) visualized by electron microscopy of PTA-stained spread preparations (from Albini and Jones 1987). **b** Leptotene/zygotene nucleus illustrates bridges containing single-strand binding protein RPA (*white arrows*), a direct player in Rad51/Dmc1-mediated strand exchange for recombination, with accompanying onset of synapsis. *Green* indicates SMC3 cohesin axis, *blue* centromeres (blue), and *red* RPA protein (from Oliver-Bonet et al. 2007). **c**, **d** Bridge configurations and incipient synapsis corresponding to the stage in **b. e** Pachytene synaptic configurations with SYCP3 axes of the SC (*red*), centromeres (*blue*), and Mlh1 foci marking sites of COs (*green*) (from Gruhn et al. 2013). **f** Two bivalents showing, respectively, extensive synapsis in progress and coalignment. In **c**, **d**, **f**, *black arrows* indicate examples of “nodules” or bridges of five types: (a) associated with SCs, (b) with association sites, (c) midway between axial cores in close alignment, (d) paired structures at matching sites on axial cores, and (e) apparently bridging the space between two converging axial cores. *Blue arrows and text* indicate positions of forming/formed SC

We have proposed a specific model for how CO patterning might occur (Fig. 4a; Kleckner et al. 2004; Wang et al. 2015 and references therein). This model, which can accurately explain chiasma patterns in a variety of diploid species (Zhang et al. 2014a, b; Wang et al. 2015), has two key features. First, each CO designation sets up an “interference signal” that spreads outward in both directions from the designation site, disfavoring the occurrence of additional COs in its path. This signal is strongest at its point of origin and dissipates in strength with an increasing distance away from that starting position. Thus, a first CO designation will set up a surrounding zone of interference. A second CO designation will tend to occur outside that zone of interference. Any subsequent COs will tend to “fill in the holes” between the previously established zones, resulting in a tendency for COs to be evenly spaced, as observed (e.g., Fig. 4e).

Second, the process of CO designation is very efficient. As one consequence of this effect, each pair of homologs will undergo at least one CO designation, thus ensuring a first obligatory CO. Thereafter, CO designations will continue to occur as long as there are still regions that have not been affected by interference (or, more precisely, where the effects of interference are not great enough to impede CO designation). Given this situation, the final array of COs will be determined by three factors: the position(s) of early recombination interactions along the chromosomes, the strength of the CO designation process in relation to the inhibitory effects of interference, and the distance over which CO interference acts. In an extreme case, where the interference distance is longer than the length of the chromosome, each homolog pair will acquire one and only one CO, regardless of the number and positions of early recombination interactions. In diploid species, this situation occurs both genome wide (e.g., *Caenorhabditis elegans*; Martinez-Perez and Colaiacovo 2009; Hillers and Villeneuve 2003) and for the shorter chromosomes in the complements of several organisms, e.g., the locust *Schistocerca gregaria* (Fox 1973).

In most organisms, including *Arabidopsis* and other plants, CO designation and interference are thought to be implemented at a particular stage of meiosis, late leptotene, when homologs are coaligned at a distance of ∼400 nm (Fig. 4b–d, f; Zhang et al. 2015; Sanchez Moran et al. 2001; C.F., unpublished). Coalignment is affected by early recombination interactions, which, in favorable cases, can be seen as “bridges” linking the structural axes of coaligned partner chromosomes (Fig. 4b–d, f; Zickler and Kleckner 2015). Notably, however, the same conclusions will pertain as long as CO designation and interference operate on the array of total DSB-mediated recombination interactions, whether specifically at the “bridge stage” or not.

### CO formation in autotetraploids: a proposal

**Fig. 5:**
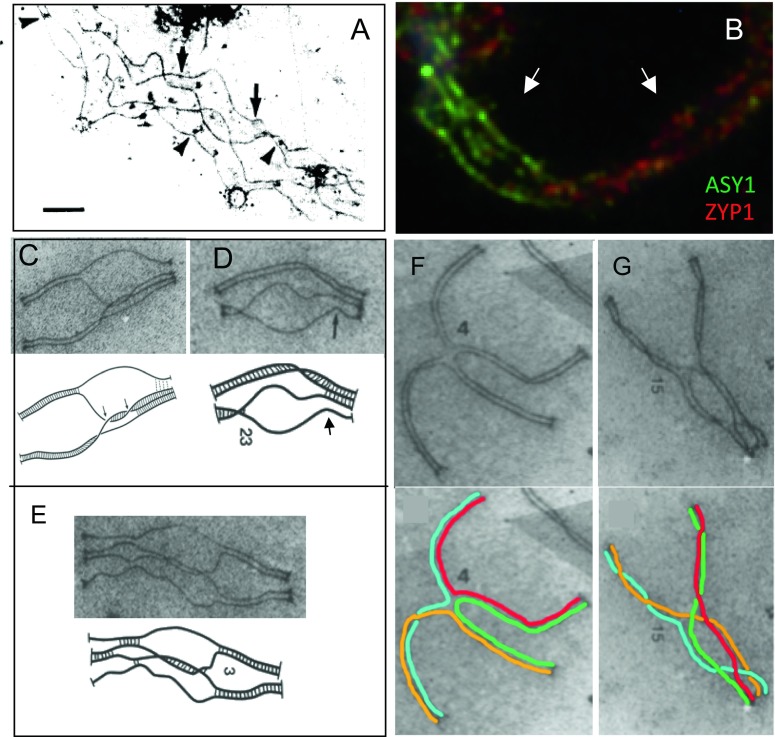
Prophase relationships among homologous chromosomes in autotetraploids. **a** Coalignment of four homologous chromosome axes at mid-prophase in tetraploid onion (*Allium porrum*) (from Stack and Roelofs 1996). The *arrows* indicate early recombination nodules which mark the sites of early recombination interactions. **b** Immunostaining of the spread *Arabidopsis arenosa* tetraploid for axis component ASY1 and SC component ZYP1, showing both coalignment of all four homolog axes (*left arrow*) and pairwise synapsis (*right arrow*) (**c**) (C. Morgan and C.F., unpublished). **c**–**g** Quadrivalents in tetraploid *Bombyx* spermatocytes (from Rasmussen 1987). **c**–**e** Three examples of configurations exhibiting partial synapsis plus pre-synaptic associations (e.g., *arrow* in **d**). **f**, **g** Two configurations exhibiting nearly complete synapsis. The four chromosomes are drawn with *different colors*. Note that chromosomes twist during SC formation. Quadrivalent frequencies diminish as the extent of synapsis increases such that, by the end of pachytene, the frequency of quadrivalents closely matches the frequency of chiasmata seen at metaphase I. By implication, the associations seen at the end of pachytene are stabilized by the occurrence of crossing over (or, at least, “crossover designation”), with one CO on each of the four arms. Once the SC disappears, these pachytene quadrivalents lead to ring quadrivalents at metaphase I

The above description of meiotic prophase would suggest that in a newly formed autotetraploid, DSBs will occur on all four chromosomes and DSB-mediated bridge interactions will occur promiscuously among all possible pairs of chromosome axes. CO designation would then be imposed on this four-chromosome array. This notion is supported by tetrasomic inheritance (above) and by studies of axis relationships in autotetraploids, where coalignment of multiple axes has been observed prior to and after synaptonemal complex (SC) formation. Such complex partner interactions are common in newly formed autotetraploids (Jones and Vincent 1994; Stack and Roelofs 1996; Carvalho et al. 2010; Rasmussen 1987; Jones and Vincent 1994; Fig. 5a–e).

**Fig. 6:**
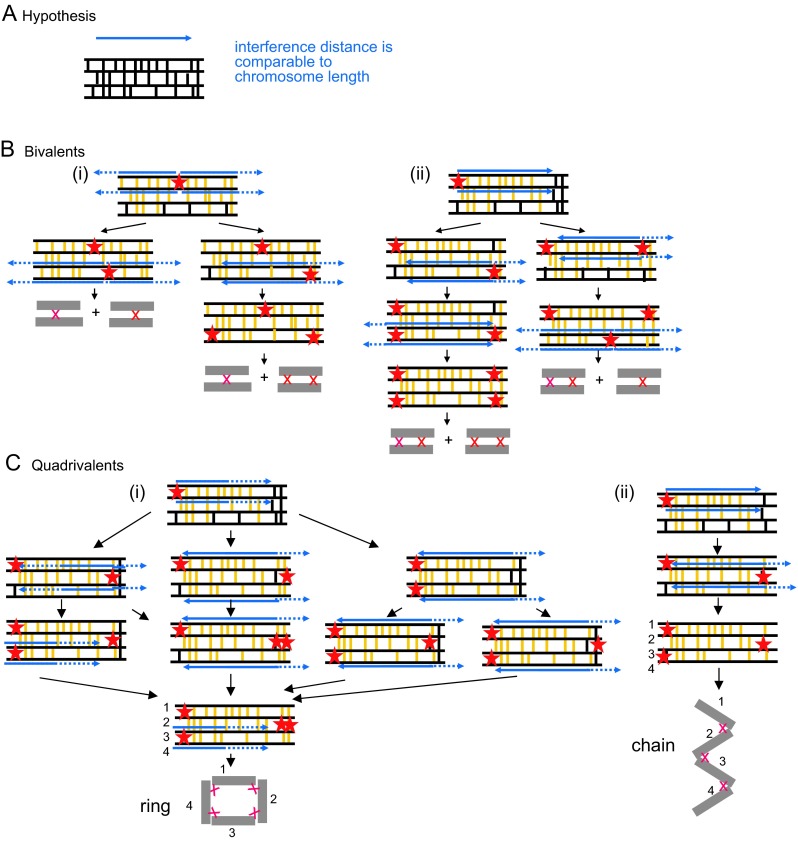
Predicted chiasma/CO patterns for an evolved autotetraploid. **a** CO sites are proposed to be selected with efficient CO designation and accompanying CO interference which extends over an effective distance comparable to the length(s) of the chromosome(s) (text; representations as in Fig. 4a). If the interference distance is longer than the chromosome length, the predicted outcome is a full complement of bivalents, each with a single chiasma (not shown). **b**, **c** Predicted outcomes if the interference distance is somewhat less than the total chromosome length comprise a mixture of bivalents and quadrivalents, where the quadrivalents are chains or rings in which all chromosomes are linked by (sub-)terminal chiasmata. **b** Bivalents of different types can arise if the first chiasma is interstitial (*i*) or sub-terminal (*ii*) and according to the starting array of early recombinational interactions (not shown). **c** Quadrivalents can only arise if the first chiasma is sub-terminal and will comprise rings (*i*) or chains (*ii*) according to the particular starting array of early recombinational interactions. In the example shown, the right-most interaction between top and bottom chromosomes is present in *i* but absent in *ii*, thereby limiting the number of CO designations to 3. Importantly, the occurrence of univalents, e.g., in trivalent-plus-univalent configurations, is precluded with efficient CO designation which ensures that every chromosome will experience at least one such event (text; not shown)

We suggest here that a critical event in the evolution of a fertile autotetraploid species is an increase in the “interference distance,” i.e., the effective distance over which the inhibitory interference signal spreads to the point where it is comparable to, or greater than, the total lengths of the chromosomes (Fig. 6a). This required effect could be achieved by directly altering the spreading interference process per se, so that inhibition extends over a greater distance from the nucleating CO site. Alternatively, given that the metric of interference is physical chromosome length (above), the equivalent effect could be achieved by shortening the physical lengths of the chromosomes: if chromosome length is decreased, a given interference distance will comprise a larger fraction of chromosome length not only in physical distance but also in genomic distance (Mb) or genetic distance (cM).

An increase to an appropriate (effective) interference distance will reduce the total number of COs/chiasmata. It will also specifically generate exactly the chiasma configurations observed in evolved autotetraploids. The effectiveness of this change is a direct consequence of the basic logic of the CO patterning process (above). The efficiency of CO designation will ensure that every chromosome copy acquires at least one CO/chiasma, thus eliminating “zero-CO” univalents in general and trivalent-plus-univalent configurations in particular. Concomitantly, operation of interference over a sufficiently long distance can ensure that no chromosome becomes connected to more than two partners and, more specifically, that such two-partner connections will occur near the ends of the chromosomes. These effects are illustrated below (Fig. 6b, c).

#### Predictions

If CO interference acts over a distance longer than the length of a particular chromosome, a first CO designation will always preclude the occurrence of a second CO designation anywhere else on either of the two participating homologs, even in regions where they are paired to other partners. A second CO designation will then occur only on one of the other two homologs. The result would be a pair of bivalents, each linked by only a single CO. In this situation, the COs in question could occur anywhere along the lengths of the chromosomes.

In a less extreme case, where the interference distance is somewhat less than the length of a chromosome, the possible outcomes will be bivalents, with either a single interstitial or two terminal chiasmata, and ring and chain quadrivalents linked by terminal chiasmata (Fig. 6b, c). These outcomes can be understood as follows:

1. If the first CO designation occurs at an interstitial location, it will preclude CO designations anywhere else on the involved chromosome pair, thus ensuring that other CO designations involve the other pair to create a pair of bivalents. The second pair might also acquire a single interstitial CO; however, if the second CO designation is near a chromosome end, the third CO designation could occur at the opposite end, generating a bivalent with a pair of terminal COs (Fig. 6b(i)).
2. If the first CO designation occurs near a chromosome end, diverse patterns can arise according to the locations of the early recombination interactions and the particular sequence of subsequent CO designations (Fig. 6b(iii), c). Univalents are excluded by efficient CO designation. Thus, the only possibilities are bivalents and quadrivalents. Moreover, since an event at one end precludes the involvement of the two affected chromosomes along most of their lengths, any second CO designation involving one or both chromosomes of the original pair will occur at the opposite end from the first CO designation. Given these rules, the only possible outcome is a pair of bivalents [at least one of which has a pair of terminal chiasmata, and the other of which may be the same or may have a single interstitial chiasma (Fig. 6b(ii)); as linkages progress from chromosome to chromosome, ring and chain quadrivalents linked by terminal chiasmata (Fig. 6c)]. Chains and rings are distinguished by the particular pattern of early recombination events linking the four chromosomes prior to CO designation (Fig. 6c(i, ii)).

It can be noted that these outcomes can emerge from situations in which the four homologous chromosomes are linked by early recombinational interactions all along their lengths. Of course, however, if early CO designations occur preferentially near the chromosome ends, then outcomes involving terminal chiasmata will be more strongly favored. A recent analysis of diploid barley raises an interesting possibility of this nature (Higgins et al. 2014b). In that species, the initiation of recombination occurs along the lengths of the chromosomes but occurs much earlier near chromosome ends than in interstitial regions. Moreover, COs/chiasmata occur differentially in the terminal regions. It was suggested that this pattern could reflect the operation of CO interference, with early CO designations in terminal regions setting up interference that spreads inward, thus precluding interstitial CO designations. Importantly, by this hypothesis, terminal chiasmata are the indirect consequence of three factors: localized recombination initiation, the temporal program of CO designation, and CO interference over an appropriate distance. There would be no intrinsic local preference for CO designation to occur at chromosome ends per se. Operation of such a system would obviously be advantageous also for autotetraploids.

We also note that a role for interference for chiasma configurations in tetraploids has been considered by Sybenga and colleagues (e.g., Sybenga et al. 1994), although not elaborated in a context of recent advances in understanding of this process.

#### Supporting evidence

Several additional observations support the above scenario.

MI bivalent associations seen in well-evolved autotetraploids often involve one and only one chiasma, versus larger numbers of chiasmata per bivalent in their diploid precursors (e.g., above; Dawson 1941; Mulligan 1967; Gillies 1969; Wolf et al. 1989; Davies et al. 1990; Carvalho et al. 2010; Yant et al. 2013).

The proposed mechanism predicts that within a given organism, when different chromosomes are of different lengths, bivalents will be more likely for shorter chromosomes whereas quadrivalents will be more likely for longer chromosomes. Correspondingly, in experimentally evolved autotetraploids of *A. thaliana* and natural polyploids of *Zea perennis* and *L. corniculatus*, the shorter chromosomes tend to occur as bivalents whereas the longer chromosomes tend to occur as quadrivalents with terminal associations (Dawson 1941; Shaver 1962; Davies et al. 1990; Santos et al. 2003). Interestingly, also, when chiasma configurations are analyzed as a function of evolutionary time after a de novo creation of an autotetraploid, regular segregation is achieved first for shorter chromosomes with longer chromosomes following later (Santos et al. 2003).

One way to increase the effective interference distance would be to decrease chromosome length (above). In accordance with this possibility, evolved autotetraploids in the three well-defined cases have been shown to have shorter prophase chromosomes than the diploids from which they evolved. This is true in the sand cress (*A. arenosa*; Higgins et al. 2014a), alfalfa (*Medicago sativa*; Gillies 1969), and male silk moths (*Bombyx mori*; Rasmussen 1987). In *Bombyx*, for example, the total diploid SC length per nucleus is 213 μm at early pachytene and 215 μm at late pachytene while the tetraploid length is 190 μm at early pachytene and 186 μm at late pachytene. Whether the actual interference distance (in μm) also increases in these situations remains to be determined.

In cereals and grasses, all COs/chiasmata are relatively terminal. Classical studies have argued for a model in which pairs of bivalent “ends” engage in CO formation randomly in all combinations (the so-called “random end pairing model,” where the word “pairing” was used to denote chiasma formation; Morrison and Rajhathy 1960a, b; discussion in Santos et al. 2003). The predicted proportion of bivalents versus quadrivalents in such case is ∼1/3 versus ∼2/3, which matches the experimental observations in these organisms (e.g., Morrison and Rajhathy 1960b). This outcome is explained explicitly by the scenario proposed above, if one further assumes a strong tendency for first CO designations to occur near chromosome ends (e.g., as in barley diploid meiosis; above).

#### Other possibilities?

What other modulations of the recombination process might allow evolution of stably fertile autotetraploid species? It might be imagined that the number of COs might be reduced in other ways. One possibility would be a reduction in the number of total recombination interactions. However, this is unlikely to be a major factor because of the phenomenon of CO homeostasis (Martini et al. 2006; Zhang et al. 2014b): as the number of total interactions decreases, the probability that a given interaction will be subject to CO interference also decreases, thereby counterbalancing the effect of fewer initial interactions. CO number could also be decreased in two other ways: first, the efficiency of CO designation could be reduced, and second, CO-designated interactions might not all mature into COs (Zhang et al. 2014a). Such changes might result, for example, from hypomorphic alterations in molecules involved specifically in the CO formation process. However, all of these possible effects also increase the probability that some chromosome pairs will not acquire even a single interaction and thus will not acquire even a single CO and thus, potentially, the frequency of univalents (Zhang et al. 2014a). In contrast, no such risk is conferred by reducing the CO number by increasing the interference distance. Additionally, these types of changes will reduce the CO formation randomly along the chromosomes and thus cannot explain changes in CO positioning.

For species that exhibit mostly terminal COs, it is alternatively possible to envision that CO designation might be intrinsically limited to regions near chromosome ends, irrespective of interference, either because early interactions are limited to these regions or because the CO designation probability is much higher in these regions. In either of these cases, and assuming that CO designation is very efficient, random CO designation at pairs of ends, in all possible combinations, could have the same effect as the model proposed here, e.g., for cereals and grasses as discussed above. However, such a model cannot explain cases in which other patterns are observed, e.g., when interstitial chiasmata are common or others. In contrast, the proposed model can synthetically explain diverse observed patterns.

It is also possible that a significant role is played by events during early prophase by which chromosomes first choose partners, i.e., by partner interactions even before DSB formation (“DSB-independent pairing”) or during DSB-mediated coalignment process prior and prerequisite to CO designation. This may seem unlikely in view of the complex patterns of associations that are seen at leptotene/zygotene in evolved tetraploid lines as well as their newly formed counterparts (e.g., Fig. 5a, b; Hobolth 1981; Gillies et al. 1987; Rasmussen 1987; Davies et al. 1990; Carvalho et al. 2010). On the other hand, in a Sordaria diploid, a mutation that permits DSB formation but delays coalignment also results in massive chromosome entanglements, implying that DSB-mediated coalignment at one position tends to promote subsequent coalignment events at adjacent positions, thus drawing the pair of partners out of the “pairing pool” (Storlazzi et al. 2010). Operation of such an effect in a tetraploid would tend to promote simpler arrays of coalignment associations and, thus, bivalent formation versus formation of multivalents. Similarly, homology-based pairwise associations in centromere regions (e.g., within non-specific centromere clusters) or early pairwise DSB-independent pairing of telomeres (review in Zickler and Kleckner 2015; Higgins et al. 2014a) might propagate along the corresponding chromosome arms, by independent pairing and/or DSB-mediated coalignment, again simplifying partner associations and, thus, CO patterns. Simplification at this early stage has a particular advantage that it would affect not only the pairwise connections resulting from COs that are subject to interference but also those resulting from the significant minority of COs that occur without being subject to CO interference (e.g., Mercier et al. 2015).

Another interesting point is that, since the two genomes of a diploid are never perfectly identical, whole genome duplication, e.g., by colchicine treatment, may produce an autotetraploid comprising two pairs of genomes where the members of each pair are identical while the members of different pairs have slight differences. Such pair-to-pair differences play an obvious determining role in evolved allopolyploids where different pairs of closely related homologs are more different from one another and, in consequence, crossovers occur preferentially between the members of each closely related pair (e.g., Holm 1986; Martín et al. 2014). The same effects could potentially be subtly significant in autotetraploids. However, if so, their effects may not be significant beyond early generations, given that evolved autotetraploids generally show tetrasomic inheritance.

#### Elimination of interlockings?

Restriction of the number and/or positions of CO designations could potentially be sufficient to eliminate the interlock problem. Interlocks arise during the DSB-mediated coalignment of homologs and, thus, prior CO designation (von Wettstein et al. 1984; Storlazzi et al. 2010). Thus, changes in CO patterns are unlikely to alter the probability with which interlocks form. In contrast, simplification of CO patterns might well facilitate the resolution of interlocks after they have formed. For this to be true, CO interactions would have to comprise the only effective linkages between homologous chromosomes and, thus, the only linkages that are preventing entangled chromosomes from assuming a regular relationship. Available evidence is consistent with this possibility. (i) Recombinational interactions that are not CO fated have progressed to the “non-crossover fate,” and the corresponding complexes are lost from the chromosome axes. (ii) Despite the fact that SC is forming during this period, SC patterns are known to undergo adjustment to give final configurations in which SC segments correspond to CO positions (e.g., in tetraploid *Bombyx*; Fig. 5f, g). Thus, apparently, SC is destabilized except at CO sites and reforms outward from those sites (which also appears to occur in allohexaploid wheat; discussion in Zickler and Kleckner 1999). Correspondingly, SC may be a permanent impediment to interlock resolution only at positions of COs, rather than globally.

## Molecular basis of evolved autotetraploidy

Recent studies provide insights into which genes came under natural selection during adaptation to whole genome duplication in a natural autopolyploid, *A. arenosa* (Hollister et al. 2012; Yant et al. 2013). *A. arenosa* has both diploid populations and a natural autotetraploid variant that is at least 15,000 years old (Arnold et al. 2015). This autotetraploid is meiotically stable and fully fertile. Also, it exhibits features that are well explained by our proposed model. All four copies of each chromosome clearly coalign and associate in zygotene and pachytene (Carvalho et al. 2010; Higgins et al. 2014a), and yet, at metaphase I, almost only bivalents are present. Further, the autotetraploid form has lower chiasma formation rates than the diploid, usually only forming one chiasma per bivalent (Comai et al. 2003; Carvalho et al. 2010; Yant et al. 2013; Higgins et al. 2014a; Fig. 1(c), right). Moreover, the axes in evolved autotetraploid lines are 5–10 % shorter than axes in closely related diploids, implying a corresponding increase in the effective interference distance as explained above (Higgins et al. 2014a; C. Morgan, C. Franklin, and K. B., unpublished).

Additional studies provide insight into which specific molecules might be involved in stabilization of autotetraploidy in *A. arenosa*. A genome scan comparing diploid and tetraploid lines showed strong evidence of selection having acted on eight genes whose functions are exerted specifically during meiosis (Hollister et al. 2012; Yant et al. 2013). The proteins encoded by these genes include chromosome axis components ASY1 and ASY3 (homologs of budding yeast Hop1 and Red1), cohesins and cohesin-associated proteins SMC3 and SYN1 (Rec8) and PDS5, and synaptonemal complex transverse filament proteins ZYP1a and ZYP1b (encoded by two tandemly duplicated genes). Mutant studies in closely related *A. thaliana* have demonstrated that mutation of these genes results in defects in recombination, including CO formation (Bai et al. 1999; Armstrong 2002; Higgins et al. 2005; De Muyt et al. 2009; Ferdous et al. 2012). In contrast, while the alleles selected in tetraploid *A. arenosa* carry mutations that have the potential to alter protein function or form, they do not appear to be loss-of-function mutations (Yant et al. 2013; Wright et al. 2015).

It is intriguing that evolution of stable autotetraploidy involves these key axis components. Alterations in axis components are known to alter the strength of CO interference (Zhang et al. 2014b; Libuda et al. 2013) and also can alter axis length (Revenkova et al. 2004; Novak et al. 2008), which will alter the effective CO interference distance as a fraction of physical and genomic chromosome length (Zhang et al. 2014b) as described above.

## Summary

How can a newly formed autotetraploid evolve to a stable sexually reproducing state, with a concomitant modulation of chiasma number and pattern? This question has been discussed for a century or more. Recent progress in the understanding and analysis of meiotic recombination opens the way to considering specific proposals for a mechanism as well as new experimental approaches. We focus here on the possibility that modulation of crossover interference, either directly and/or indirectly via changes in axis length, could play a central role. Our proposal explains the patterns of COs/chiasmata observed for species of evolved autotetraploids that exhibit primarily bivalents and for species that exhibit both bivalents and quadrivalents as well as accommodating the tendency, seen in several cases, for COs/chiasmata to occur near chromosome ends. It may also explain the fact that evolved autotetraploids lack interchromosomal interlocks characteristic of neo-autotetraploid lines. Evaluation of this and other proposals by application of molecular methodologies provides a fertile ground for future studies.

## Electronic supplementary material

Below is the link to the electronic supplementary material.

ESM 1 (DOCX 22 kb)

ESM 2 (DOCX 733 kb)
