# Supplementary material for: The challenge of evolving stable polyploidy: could an increase in “crossover interference distance” play a central role?
Source: Chromosoma. 2016 Jan 12;125:287–300. doi: 10.1007/s00412-015-0571-4 (PMC4830878; doi:10.1007/s00412-015-0571-4)
Supplement: Supplementary file 1 — (DOCX 22 kb) [file 412_2015_571_MOESM1_ESM.docx]

**TABLE S1**

**For *Chromosoma***

Kirsten Bomblies, Gareth Jones, Chris Franklin, Denise Zickler and Nancy Kleckner^*^ **The challenge of evolving stable polyploidy: could crossover interference play a central role?**

*Coresponding author: Department of Molecular and Cellular Biology, Harvard University, Cambridge, MA, USA, kleckner@fas.harvard.edu

**TABLE S1. Cytological descriptions of evolved autotetraploids**

| **Species** | **Metaphase I Cytology (Natural)** | **References** |
| --- | --- | --- |
| *Allium porrum / ampeloprasum* | Primarily bivalents; chiasmata located proximally, adjacent to centromeres | (Levan 1940; Jones et al. 1996; Stack & Roelofs 1996) |
| *Allium vineale* | Mostly rod bivalents | (Loidl 1986) |
| *Arabidopsis arenosa* | Almost only bivalents; chiasma number ~1/bivalent. Pachytene SCs shorter in 4X than 2X. Neo-4X high multivalent frequency / low fertility. | (Comai et al. 2003; Carvalho et al. 2010; Hollister et al. 2012; Yant et al. 2013; Higgins et al. 2014.) |
| *Vaccinium corymbosum* | Primarily bivalents, occasional quadrivalents, univalents very rare | (Jelenkovic & Hough 1970; Krebs & Hancock 1989) |
| *Actinidia chinensis*  *(A. arguta)* | Mostly rod bivalents; short chromosomes; ~1.25 chiasmata per bivalent. Neopolyploid high multivalent frequency and low fertility. | (McNeilage & Considine 1989; Wu et al. 2013) |
| *Heuchera grossulariifolia* | Mostly bivalents, some quadrivalents; Low chiasma number, short chromosomes. | (Wolf et al. 1989) |
| *Lotus corniculatus* | Mostly rod bivalents, some univalents. Quadrivalents occur preferentially w/larger chromosomes & w/terminal chiasmata; chiasma # =1.13 / 2 chrom. | (Dawson 1941; Davies et al. 1990) |
| *Medicago sativa* | Almost only bivalents. Chiasmata usually near centromere or interstitial. Pachytene chromosomes shorter than in 2X. | (Gillies 1969; Armstrong 1971; Quiros 1982) |
| *Physaria vitulifera* | 2X slightly more than 2 chiasmata per bivalent; 4X uniformly single chiasma near center of chromosome. | (Mulligan 1967) |
| *Tolmeia menziesii* | Mostly bivalents. | (Soltis & Rieseberg 1986) |
| *Zea perennis* | Some quadrivalents, but fewer than in 4X maize, lower chiasma number than 4X maize. 39% of bivalents open, only 12% in 4X maize. | (Shaver 1962) |
| *Chrysanthemum yoshinaganthum* | Mostly bivalents, more rod than ring bivalents. | (Tanaka 1960) |
| *Sorghum spp.* | 5 species natural 4X - fewer multivalents & univalents, more rod bivalents than neopolyploids. | (Reddi 1970) |
| *Dioscorea alata* | 1/3 chrom associate in quadrivalents, rest in bivalents; no uni- or tri-valents; Quadrivalents segregate normally. | (Abraham et al. 2013) |
| *Chamerion angustifolium* | Some populations mostly bivalents, others up to 35% chromosomes in quadrivalents. Almost all quadrivalents rings with alternate disjunction. | (Mosquin 1967) |

| *Dactylis omerata* | Quadrivalents common; chiasmata primarily terminal; quadrivalents mostly chains or rings; uni- and tri-valents rare. Neopolyploid has lower chiasma number than natural 4X, lower quadrivalent frequency and more uni- and trivalents. | (McCollum 1958) |
| --- | --- | --- |

**Table References**

Abraham K, Nemorin A, Amau G (2013) Meiosis and sexual fertility of autotetraploid clones of greater yam *Dioscorea alata* L. Genet Resour Crop Evol 60:819–823.

Armstrong KC (1971) Chromosome associations at paachytene and metaphase in *Medicago sativa*. Can J Genet Cytol 13:697–702.

Carvalho A, Delgado M, Barão A, et al. (2010) Chromosome and DNA methylation dynamics during meiosis in the autotetraploid Arabidopsis arenosa. Sex Plant Reprod 23:29–37. doi: 10.1007/s00497-009-0115-2

Comai L, Tyagi AP, Lysak MA (2003) FISH analysis of meiosis in *Arabidopsis* allopolyploids. Chromosome Res 11:217–226.

Davies A, Jenkins G, Rees H (1990) Diploidization of *Lotus corniculatus* L. (Fabaceae) by elimination of multivalents. Chromosoma 99:289–295.

Dawson C (1941) Tetrasomic inheritance in *Lotus corniculatus* L. J Genet 42:49–73.

Gillies CB (1969) Alfalfa chromosomes. II. Pachytene karyotype of a tetraploid *Medicago sativa* L. Crop Science 10:172–175.

Higgins JD, Wright KM, Bomblies K, Franklin FCH (2014) Cytological techniques to analyze meiosis in Arabidopsis arenosa for investigating adaptation to polyploidy. Front Plant Sci 4:546. doi: 10.3389/fpls.2013.00546

Hollister JD, Arnold BJ, Svedin E, et al. (2012) Genetic adaptation associated with genome-doubling in autotetraploid Arabidopsis arenosa. PLoS Genet 8:e1003093. doi: 10.1371/journal.pgen.1003093

Jelenkovic G, Hough LF (1970) Chromosome associations in the first meiotic division in three tetraploid clones of *Vaccinium corymbosum* L. Can J Genet Cytol 12:316–324.

Jones GH, Khazanehdari KA, Ford-Lloyd, BV (1996) Meiosis in the leek (*Allium porrum* L.) revisited. II. Metaphase I observations. Heredity 76:186–191.

Krebs SL, Hancock JF (1989) Tetrasomic inheritance of isoenzyme markers in the highbush blueberry *Vaccinium corymbosum* L. Heredity 63:11–18.

Levan A (1940) Meiosis of *Allium porrum*, a tetraploid species with chiasma localisation. Hereditas 26:454–462.

Loidl J (1986) Synaptonemal complex spreading in *Allium*. II. Tetraploid *A. vineale*. Can J Genet Cytol 28:754–761.

McCollum CD (1958) Comparative studies of chromosome pairing in natural and induced tetraploid Dactylis. Chromosoma 9:571–605.

Mcneilage MA, Considine JA (1989) Chromosome studies in some Actinidiataxa and implications for breeding. New Zealand Journal of Botany 27:71–81. doi: 10.1080/0028825X.1989.10410145

Mosquin T (1967) Evidence for Autopolyploidy in *Epilobium angustifolium* (Onagraceae). Evolution 21:713–719.

Mulligan A (1967) Diploid and tetraploid *Physaria vitulifera* (Cruciferae). Can J Bot 45:183–188.

Quiros CF (1982) Tetrasomic segregation for multiple alleles in alfalfa. Genetics 101:117–127.

Reddi VR (1970) Pachytene pairing and the nature of polyploidy in *Sorghum arundinaceum*. Caryologia 23:295–302.

Shaver DL (1962) A Study of Meiosis in Perennial Teosinte, in Tetraploid Maize and in their Tetraploid Hybrid. Caryologia 15:43–57.

Soltis PS, Rieseberg L (1986) Autopolyploidy in *Tolmiea menziesii* (Saxifragaceae): Genetic insights from enzyme electrophoresis. Am J Bot 73:310–318.

Stack SM, Roelofs D (1996) Localized chiasmata and meiotic nodules in the tetraploid onion *Allium porrum*. Genome 39:770–783. doi: 10.1139/g96-097

Tanaka R (1960) On the speciation and karyotypes in diploid and tetraploid species of Chrysanthemum V. Chrysanthemum Yoshinaganthum (2n=36). Cytologia 25:43–58.

Wolf PG, Soltis PS, Soltis DE (1989) Tetrasomic inheritance and chromosome pairing behaviour in the naturally occurring autotetraploid *Heuchera grossulariifolia* (Saxifragaceae). Genome 32:655–659. doi: 10.1139/g89-494

Wu J-H, Datson PM, Manako KI, Murray BG (2013) Meiotic chromosome pairing behaviour of natural tetraploids and induced autotetraploids of *Actinidia chinensis.* Theor Appl Genet 127:549–557. doi: 10.1007/s00122-013-2238-y

Yant L, Hollister JD, Wright KM, et al. (2013) Meiotic adaptation to genome duplication in *Arabidopsis arenosa.* Curr Biol 23:2151–2156. doi: 10.1016/j.cub.2013.08.059
