# Supplementary material for: The challenge of evolving stable polyploidy: could an increase in “crossover interference distance” play a central role?
Source: Chromosoma. 2016 Jan 12;125:287–300. doi: 10.1007/s00412-015-0571-4 (PMC4830878; doi:10.1007/s00412-015-0571-4)
Supplement: Supplementary file 2 — (DOCX 733 kb) [file 412_2015_571_MOESM2_ESM.docx]

**For *Chromosoma***

Kirsten Bomblies, Gareth Jones, Chris Franklin, Denise Zickler and Nancy Kleckner^*^ **The challenge of evolving stable polyploidy: could crossover interference play a central role?**

*Corresponding author: Department of Molecular and Cellular Biology, Harvard University, Cambridge, MA, USA, [kleckner@fas.harvard.edu](mailto:kleckner@fas.harvard.edu)


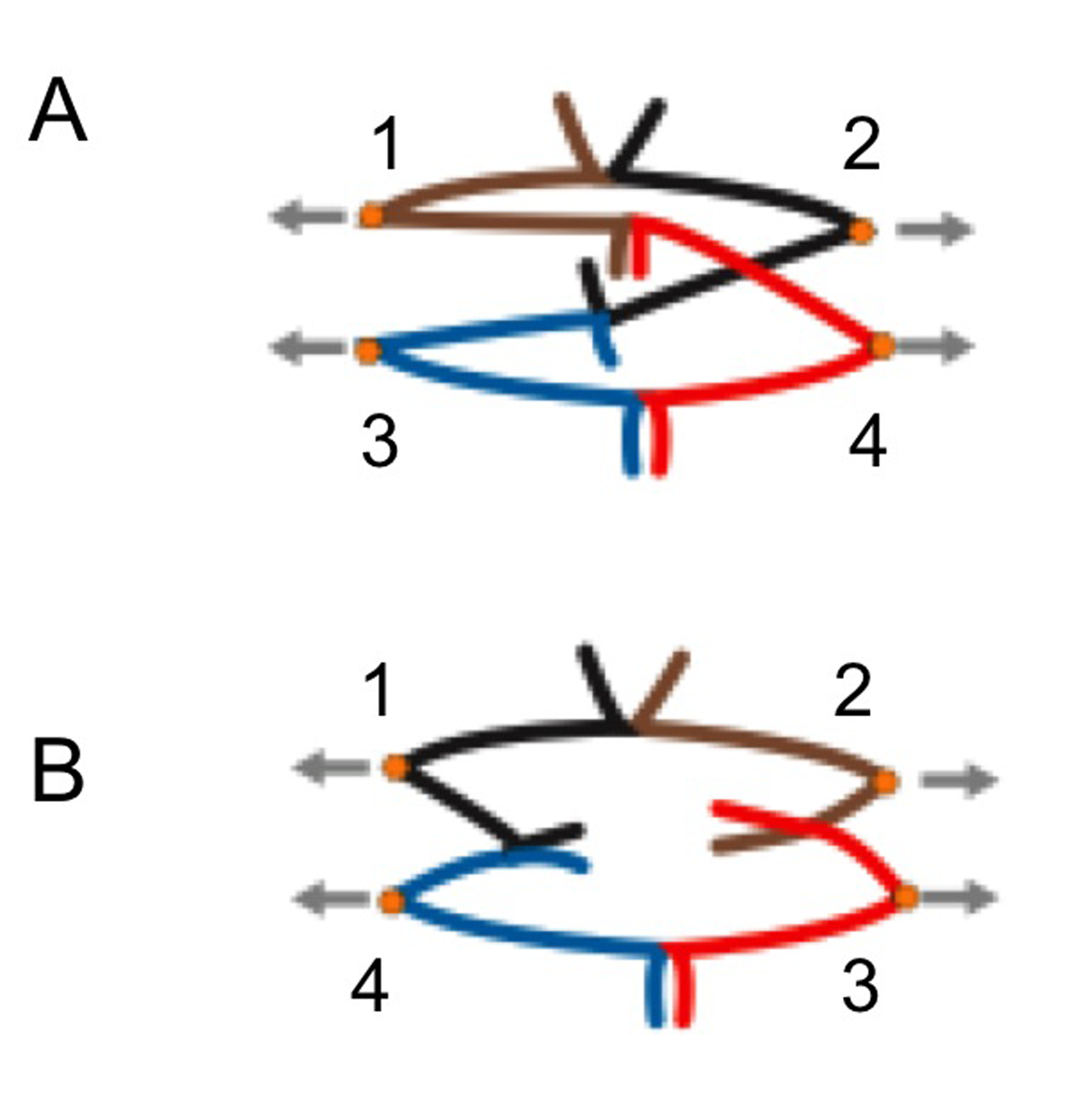


**Figure S1.** Two MI chiasma configurations effective for segregation of a ring quadrivalent.

(A) Centromeres around the ring are oriented to the two poles in alternation: centromeres 1 and 3 to one pole; and centromeres 2 and 4 to the other pole. (B) Two centromeres at adjacent positions in the ring are oriented to one pole (1,4) while the other two, also adjacent, are oriented to the other pole (2,3).

*Note: These configurations are classically referred to as "alternate" and "adjacent". In (A), "alternate" centromeres are oriented to the same pole (i.e. 1 vs 2, 2 vs 3, 3 vs 4 and 4 vs 1). In (B), (some pairs of) "adjacent" centromeres are oriented to the same pole (i.e.. 2+3 and 1+4).*

In (A), all four centromeres are under tension by pulling forces exerted from both sides. For example, cen 1 is under tension via chiasma linkages to both cen 2 and cen 4.

In (B), all four centromeres are under tension by pulling forces exerted from only one direction. For example, cen 1 is under tension via chiasma linkage to cen 2, but not via chiasma linkage to cen 4, which is oriented towards the same pole.

As a result, (A) is expected to be better discriminated from other configurations than (B). This difference may underlie the fact that as autotetraploid segregation increases in fidelity, the relative abundance of (A) increases relative to (B) (McCollum 1958; Mosquin 1967).

It has also been noted that there is a genetically-based association between the stability of quadrivalent segregation and the occurrence of terminal chiasmata. (Myers 1945; McCollum 1958; Hazarika and Rees 1967; Jones 1967). Perhaps terminal localization of chiasmata is favored because, by providing a longer "tether" between adjacent centromeres, it facilitates alternate orientation of adjacent centromeres to opposite poles (configuration A), thus giving optimally regular segregation.

Terminal localization of chiasmata could result from either of two effects (not mutually exclusive). First, as described above, it might arise from an array of early recombination intermediates that are relatively well-spread throughout the chromosomes by the effects of interference (e.g. text Figure 5, right side). However, in several cases of strongly terminal chiasma localization in diploids, this pattern can be attributed to terminal localization of recombination-initiating DSBs (and thus COs/chiasmata) (e.g. Higgins et al., 2014; Viera et al., 2010).

**References**

Hazarika MH, Rees H (1967) Genotypic control of chromosome behaviour in rye. X. Chromosome pairing and fertility in autotetraploids. Heredity 22:317–322. doi: 10.1038/hdy.1967.44

Higgins JD, Osman K, Jones GH and Franklin FC (2014) Factors underlying restricted crossover localization in barley meoisis. Ann Ref Genet 48:29-47.

Jones GH (1967) The control of chiasma distribution in rye. Chromosoma 22:69–90. doi: 10.1007/BF00291287

McCollum CD (1958) Comparative studies of chromosome pairing in natural and induced tetraploid Dactylis. Chromosoma 9:571–605.

Mosquin T (1967) Evidence for Autopolyploidy in *Epilobium angustifolium* (Onagraceae). Evolution 21:713–719.

Myers WM (1945) Meiosis in autotetraploid *Lolium perenne* in relation to chromosomal behavior in autopolyploids. Bot Gaz 106:304–316.

Viera A, Santos JL, Parra MT, Calvente A, Gomez R, de la Fuente R, Suja JA, Page J, de la Vega CG, Rufas JS. (2010) Incomplete synapsis and chiasma localization: the chicken or the egg? Cytogenet Genome Res 128:139-151. doi: 10.1159/000290637.
